# Short‐term water deprivation attenuates the exercise pressor reflex in older female adults

**DOI:** 10.14814/phy2.14581

**Published:** 2020-09-23

**Authors:** Joseph C. Watso, Austin T. Robinson, Matthew C. Babcock, Kamila U. Migdal, Melissa A. H. Witman, Megan M. Wenner, Sean D. Stocker, William B. Farquhar

**Affiliations:** ^1^ Department of Kinesiology and Applied Physiology University of Delaware Newark DE USA; ^2^ School of Kinesiology Neurovascular Physiology Laboratory Auburn University Auburn AL USA; ^3^ Department of Medicine University of Pittsburgh Pittsburgh PA USA

**Keywords:** aging, exercise pressor reflex, hypohydration

## Abstract

Older adults have reduced fluid intake and impaired body fluid and electrolyte regulation. Older female adults exhibit exaggerated exercise blood pressure (BP) responses, which is associated with an increased risk of adverse cardiovascular events. However, it is unclear if dysregulated body fluid homeostasis contributes to altered exercise BP responses in older female adults. We tested the hypothesis that short‐term water deprivation (WD) increases exercise BP responses in older female adults. Fifteen female adults (eight young [25 ± 6 years] and seven older [65 ± 6 years]) completed two experimental conditions in random crossover fashion; a euhydration control condition and a stepwise reduction in water intake over three days concluding with a 16‐hr WD period. During both trials, beat‐to‐beat BP (photoplethysmography) and heart rate (electrocardiogram) were continuously assessed during rest, handgrip exercise (30% MVC), and post‐exercise ischemia (metaboreflex isolation). At screening, older compared to young female adults had greater systolic and diastolic BP (*p* ≤ .02). Accelerometer‐assessed habitual physical activity was not different between groups (*p* = .65). Following WD, 24‐hr urine flow rate decreased, whereas thirst, urine specific gravity, and plasma osmolality increased (condition: *p* < .05 for all), but these WD‐induced changes were not different between age groups (interaction: *p* ≥ .31 for all). Resting systolic and diastolic BP values were higher in older compared to young adults (*p* < .01 for both), but were not different between experimental conditions (*p* ≥ .20). In contrast to our hypothesis, WD was associated with attenuated systolic BP responses during handgrip exercise (post hoc: *p* < .01) and post‐exercise ischemia (post hoc: *p* = .03) in older, but not young, female adults. These data suggest that reduced water intake‐induced challenges to body fluid homeostasis do not contribute to exaggerated exercise BP responses in post‐menopausal female adults.

## Graphical abstract

The current data suggest that healthy female aging is associated with a maintained ability to preserve body water balance during short‐term reductions in water intake. These data also indicate that reduced water intake is not associated with augmented cardiovascular responses during exercise pressor reflex activation in older female adults.

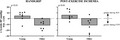

**What is the central question of this study?**

Compared to young adults, older adults tend to have reduced fluid intake and impaired body fluid regulation. Post‐menopausal female adults exhibit the largest exercise blood pressure (BP) responses of any age or sex group, which is associated with an increased risk of future cardiovascular events. However, it is unclear if dysregulated body fluid homeostasis contributes to augmented exercise BP responses in post‐menopausal female adults.

**What is the main finding and its importance?**

We found that short‐term water deprivation reduces exercise BP responses in healthy post‐menopausal female adults. These data suggest that reduced water intake may not contribute to exaggerated exercise BP responses in older female adults.

## 1 INTRODUCTION

Cardiovascular disease is the leading cause of death among adults in the United States (Xu, Murphy, Kochanek, Bastian, & Arias, 2018) and worldwide (Global & Estimates, 2016). Increasing age (Xu et al., 2018), high resting arterial blood pressure (BP) (i.e., hypertension) (Virani et al.,), and exaggerated exercise BP responses (Matthews, Woodall, & Allen, 1993; Miyai et al., 2002; Schultz, Otahal, Picone, & Sharman, 2015; Tzemos, Lim, Mackenzie, & MacDonald, 2015) are associated with greater future risk for developing cardiovascular disease. Several prior studies have demonstrated that older adults experience augmented BP responses during static and dynamic exercise compared to young adults (Boutcher & Stocker, 1999; Choi et al., 2012; Hartog, Bolignano, Sijbrands, Pucci, & Mattace‐Raso, 2018; Lalande, Sawicki, Baker, & Shoemaker, 2014; Milia et al., 2015). More recent work suggests that older female adults have the largest BP responses compared to other age and sex groups (Trinity, Layec, Hart, & Richardson, 2018). Thus, there is a critical need to determine what physiological factors contribute to BP dysregulation during exercise in older female adults, a growing (Heidenreich et al., 2011), and understudied part of the population.

Chronic hypohydration is associated with a greater future incidence of cardiovascular disease (Chan, Knutsen, Blix, Lee, & Fraser, 2002), although the mechanisms underlying this relation are unclear (Watso & Farquhar, 2019). Older adults are more commonly underhydrated (Drewnowski, Rehm, & Constant, 2013) and have a greater incidence of plasma hypertonicity (i.e., high basal plasma osmolality) (Stookey, 2005). Additionally, old, compared to young, adults have augmented increases in plasma osmolality following short‐term water deprivation (WD) (Phillips et al., 1984). This is likely because older adults are not able to regulate body water balance as well as their younger counterparts because of reduced thirst sensations (Phillips, Bretherton, et al., 1993; Phillips et al., 1984), lower total body water (Davy & Seals, 1985), altered extracellular fluid sensing (Phillips, Johnston, & Gray, 1993), blunted hormonal (e.g., antidiuretic hormone) release (Bevilacqua et al., 1987; Phillips, Bretherton, et al., 1993), and impaired kidney function (Crowe et al., 1987). However, the effects of short‐term WD on reflex BP regulation during exercise in older adults remains unknown.

WD elevates sympathetic nerve activity to support BP in rodents (Brooks, Freeman, & Clow, 2004; Brooks, Qi, & O'Donaughy, 2005). However, WD does not alter the reflex regulation of BP in rodents or healthy young adults. For example, 48‐hr of WD in male rats did not exaggerate BP responses to unilateral microinjection of glutamate (or GABA) in the rostral ventrolateral medulla (RVLM) or during the stimulation of sciatic afferents. Consistent with these findings in rodents, we demonstrated that short‐term WD does not increase exercise BP responses in healthy young male and female adults (Watso et al., 2019). However, prior literature indicates that older compared to young male adults have greater increases in plasma osmolality and blood electrolyte concentrations following WD (Phillips et al., 1984), which could exaggerate BP responses during exercise in older adults. Thus, we hypothesized that older versus young female adults would also have greater increases in plasma osmolality following short‐term WD and would consequently demonstrate augmented BP responses during isometric handgrip exercise (HG), metaboreflex isolation (post‐exercise ischemia; PEI), and the cold pressor test (CPT). Our reasoning was that the hypothesized larger increases in plasma osmolality and blood electrolyte concentrations in older female adults could act centrally to elicit augmented BP responses during exercise.

## 2 MATERIALS AND METHODS

### 2.1 Participants

The Institutional Review Board at the University of Delaware approved this protocol and the study conformed with the Declaration of Helsinki. The data reported here are part of a larger registered trial (ClinicalTrials.gov Identifier: NCT03560869). All participants provided verbal and written consent prior to enrollment in the study. During the initial screening visit, participants completed a physical activity readiness questionnaire, medical history questionnaire, and underwent measurements of body height and mass. Resting brachial BP was measured via auscultation in triplicate with participants in the seated position following at least five minutes of quiet rest. The inclusion criteria for this study included: age between 20 and 35 or 55 and 75 years, resting systolic BP between 90 and 140 mmHg, resting diastolic between BP 50 and 90 mmHg, and body mass index < 30 kg/m^2^ at screening. Study participants were also free of any known overt cardiovascular disease as well as neurological, renal, and pulmonary diseases. Exclusion criteria included current or recent (within past 12 months) nicotine use, previous diagnosis of hypertension, and past or current use of antihypertensive medications.

We initially enrolled 19 older adults in this study. Data from seven older adults are reported in the current manuscript because six individuals were excluded after screening, three individuals dropped out following screening, while noting low time availability and/or a loss of interest in participating, data from one individual are excluded due to an equipment malfunction, one individual was excluded because she started taking anti‐hypertensive medications prior to completing both experimental trials, and one individual had an unrelated back injury causing her to drop out of the study prior to the experimental trials. All seven older female adults were post‐menopausal (self‐report). Data from eight young adults are included, matched with the older cohort for body mass, body mass index, and physical activity habits. All participants were permitted to continue medication usage throughout the study, which included multivitamins (*n* = 2 young, *n* = 2 older), atorvastatin (*n* = 2 older), calcium supplements (*n* = 1 young, *n* = 1 older), Vitamin‐D3 (*n* = 1 older), B‐vitamin complex (*n* = 1 young), loratadine (*n* = 1 older), cranberry pills (*n* = 1 older), ibandronate (*n* = 1 older), and probiotics (*n* = 1 older).

### 2.2 Hydration conditions

**FIGURE 1:**
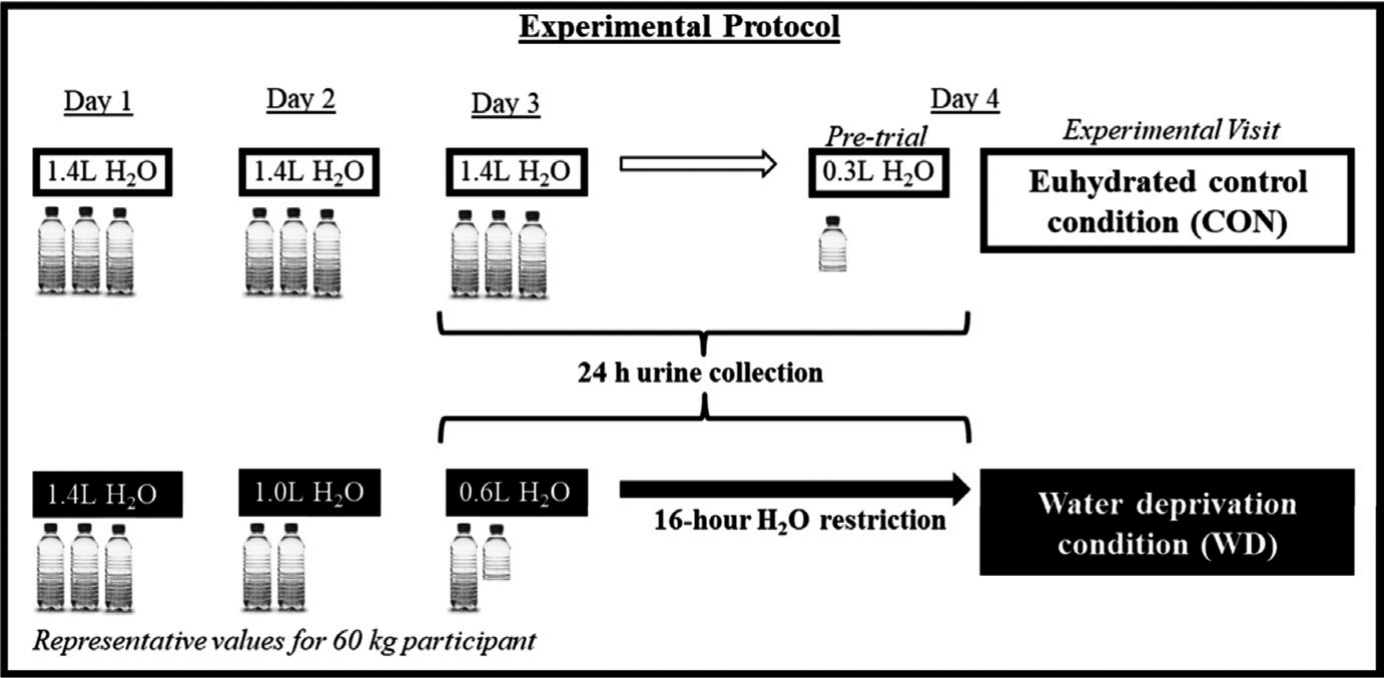
In random crossover fashion, 15 female participants completed both a euhydrated control (CON) protocol and a water deprivation (WD) protocol prior to laboratory testing on day four. In the laboratory, we assessed beat‐to‐beat blood pressure and heart rate during a handgrip exercise trial and cold pressor test

A timeline of the experimental protocol is presented in Figure 1. Participants completed two three‐day‐long hydration conditions in random order, as previously described (Watso et al., 2019). During the normal hydration condition (CON), participants were asked to consume 23 ml of H_2_O/kg body mass/day for days one through three and to consume 250 ml of H_2_O before arriving at the laboratory for testing on day four. The WD condition required participants to consume 23 ml of H_2_O/kg body mass on day one, 17 ml of H_2_O/kg body mass on day two, and 10 ml of H_2_O/kg body mass on day three, followed by a 16‐hour water abstention period prior to testing on day four. Experimental visits were separated by at least one week for older female adults and young female participants were tested in the early follicular phase of the menstrual cycle (*n* = 6; self‐report) or placebo phase of oral contraceptives (*n* = 2; self‐report). This intervention was chosen to maximize ecological validity (voluntary dehydration) and to minimize confounding effects from exercise‐, heat‐, or diuretic‐induced dehydration methods. Participants were asked to refrain from caffeine, alcohol, vigorous exercise, and exercise in the heat on days two and three during both three‐day hydration protocols. Participants were given guidance on estimating food portion sizes and keeping a diet log to maintain the recommended daily sodium intake (2,300 mg/day) (Panel on Dietary Reference Intakes for Electrolytes and Water, and Standing Committee on the Scientific Evaluation of Dietary Reference Intake, 2004). They were also instructed to consume the same foods and maintain the same physical activity during the second three‐day hydration condition as reported in their diet log during the first three‐day hydration condition. Participants collected their urine in a sterile, light protected collection container for 24 hours preceding in‐lab testing and reported to the laboratory for testing on the fourth day of each condition following a four hour fast.

### 2.3 Experimental visit

Upon arrival to the laboratory, participants provided a spot urine sample, were weighed (Tanita Body Composition Analyzer, Model TBF‐300A; Arlington Heights, IL), and provided a subjective rating of their thirst and mouth dryness using a Likert scale. The overall thirst rating was generated by calculating the mean value from the subjective rating of (a) thirst and (b) dryness of mouth on a 10 cm line with anchor points of “not at all” [left] to “very” [right]. The maximal voluntary contraction was determined using the average handgrip force produced (Grip Force Transducer, ADInstruments, Colorado Springs, CO) during three maximal forearm contractions, separated by ≥one minute each. Participants were then outfitted with the required equipment for the measurement of beat‐to‐beat BP, brachial BP, and heart rate (described below).

Participants rested quietly for 10 minutes in a dimly lit, temperature‐controlled room (22–24 °C). Following baseline data collection, participants performed a 2‐minute bout of isometric HG with their dominant hand at 30% of maximal voluntary contraction, using real‐time visual feedback of force production. Immediately prior to the cessation of HG exercise, post‐exercise ischemia (PEI) was achieved on the same arm by rapidly inflating an occlusion cuff (Hokanson, Inc, Bellevue, WA, USA) around the upper arm to 250 mmHg for three minutes to isolate the metaboreflex component of the exercise pressor reflex. Following the HG exercise trial, participants recovered for 10–15 minutes. Following a two‐minute baseline, a CPT was performed for two minutes by submerging the participants’ dominant hand in an ice‐water bath (4°C) for two minutes.

### 2.4 Cardiovascular measures

Heart rate was continuously measured using standard single‐lead ECG (Dash 2000; GE Medical Systems, WI, USA). Beat‐to‐beat arterial BP and Modelflow‐derived cardiac output (Sugawara et al., 2003; Vaal, de Wilde, van den Berg, Schreuder, & Jansen, 2005) were measured at the finger of the participants' non‐dominant hand using photoplethysmography (Finometer; Finapres Medical Systems, the Netherlands) (Guelen et al., 2003). Brachial BP was measured using an automated oscillometric sphygmomanometer (Dash 2000; GE Medical Systems, WI, USA) and used to verify absolute beat‐to‐beat BP values. Stroke volume was then calculated by dividing cardiac output by heart rate. Systemic vascular resistance (SVR) was calculated by dividing mean BP by cardiac output.

### 2.5 Blood & urine analysis

Participants laid in the supine position for ≥20 minutes prior to venous blood sample collection. Paired resting biochemical data are reported from all seven older adults and five of eight young participants due to difficulties collecting blood samples. Venous blood samples and 24‐hour urine samples were analyzed for serum and urine electrolyte concentrations (EasyElectrolyte Analyzer; Medica, Bedford, MA, USA), as well as plasma and urine osmolality (3D3 Osmometer; Advanced Instruments, Norwood, MA). Venous blood samples were also analyzed for Hb (Hb 201+; HemoCue, Lake Forest, CA, USA) and Hct (Pre‐calibrated Clay Adams, Readacrit Centrifuge; Becton Dickinson, Sparks, MD, USA). Change in plasma volume (expressed as a percentage) was calculated using the following equation (Dill & Costill, 1974):

$$\text{Plasma volume}\left(\%\right)=\left(\left(100\times \left({\text{Hb}}_{\text{CON}}/{\text{Hb}}_{\text{WD}}\right)\right)\times \left(1‐\left({\text{Hct}}_{\text{WD}}/100\right)\right)\right)/\left(\left(1‐\left({\text{Hct}}_{\text{CON}}/100\right)\right)‐100\right).$$

Urine specific gravity was determined from the spot and 24‐hour urine samples. Young female participants’ spot urine samples were also used to confirm that they were not pregnant (hCG cassettes, Moore Medical).

### 2.6 Physical activity monitoring

Habitual physical activity was objectively assessed during seven consecutive days following the second randomized experimental visit. Average daily step count and moderate‐to‐vigorous physical activity duration were determined from validated (Plasqui & Westerterp, 2007) accelerometers (ActiGraph wGT3X‐BT, Pensacola, FL, USA) after the confirmation of wear and non‐wear times based on physical activity logs and software (ActiLife v6.13.4) thresholds.

### 2.7 Statistical analysis

Cardiovascular data were collected continuously at a sampling rate of 20,000 Hz, aside from ECG which was collected at 1,000 Hz, using LabChart (LabChart 8.0 Pro, ADInstruments, Colorado Springs, CO, USA). Screening measures, changes in body mass, and changes in plasma volume between conditions were compared using unpaired, two‐tailed *t* tests. Biochemical data and resting cardiovascular measures were compared using two‐way ANOVAs (age group × condition). Cardiovascular measures collected throughout the final minute of HG, PEI, and CPT were compared to their respective baseline. BP responses during the final minute of HG, PEI, and CPT were compared using two‐way ANOVAs (age group x condition). Tukey multiple comparison testing was employed in all post hoc analyses. Finally, systolic BP responses between conditions (WD minus CON) were compared between age groups using unpaired, two‐tailed *t* tests. All data were analyzed using GraphPad Prism 8.3 (GraphPad Software Inc., La Jolla, CA) and significance was set a priori at *p* < .05. All data are presented as means ± *SD*.

## 3 RESULTS

Participant screening characteristics and objectively assessed physical activity habits are provided in Table 1. Participant reported dietary sodium consumption was similar between age groups and experimental conditions (Young CON 2,227 ± 222 vs. WD 2,193 ± 400; Older CON 2,093 ± 333 vs. WD 2,104 ± 393 mg/day; two‐way ANOVA age: *p* = .39, condition: *p* = .85, interaction: *p* = .56).

**TABLE 1:** Participant screening measures

Characteristic	Young	Older	*p*‐value
Number of female adults	8	7	
Age, yrs	25 ± 6	65 ± 6*	<.01
Body mass, kg	60 ± 12	60 ± 9	.98
Body mass index, kg • m^−2^	23 ± 4	23 ± 3	.85
Systolic BP, mmHg	104 ± 9	119 ± 13*	.02
Diastolic BP, mmHg	60 ± 7	76 ± 7*	<.01
Step count, steps • day^−1^	7,908 ± 3,135	7,505 ± 3,134	.83
MVPA, min • day^−1^	74 ± 34	64 ± 37	.65

Note

Data are presented as mean ± *SD*.

BP, arterial blood pressure; MVPA, moderate‐to‐vigorous physical activity.

* Indicates significance.

### 3.1 Hydration status following water deprivation

Short‐term WD reduced 24‐hour urine flow rate, increased 24‐hour urine osmolality, and increased 24‐hour and spot urine specific gravity (Table 2). Absolute body mass values were not different between age groups or conditions (Young CON 60.9 ± 13.3 vs. WD 60.6 ± 13.4; Older CON 59.0 ± 8.8 vs. WD 58.5 ± 8.9 kg; age: two‐way ANOVA *p* = .74, condition: *p* = .09, interaction: *p* = .66). There were no significant age‐related differences with the urinary hydration markers. Short‐term WD increased plasma osmolality and thirst rating in both age groups. Plasma osmolality was significantly higher in older compared to young adults during both conditions (Table 2). Hemoglobin concentrations (Young CON 12 ± 1 vs. WD 13 ± 1; Older CON 13 ± 1 vs. WD 13 ± 1 mg/dl; two‐way ANOVA age: *p* = .13, condition: *p* = .70, interaction: *p* = .53) and hematocrit values (Young CON 39 ± 4 vs. WD 38 ± 4; Older CON 41 ± 1 vs. WD 41 ± 2%; two‐way ANOVA age: *p* = .13, condition: *p* = .70, interaction: *p* = .53) were not different between conditions or age groups. Changes in plasma volume from CON to WD were not different between age groups (Young −1 ± 11 vs. Older 0 ± 5%, *p* = .84). Serum sodium concentrations were not different between age groups or conditions (Young CON 142 ± 1 vs. WD 141 ± 1; Older CON 143 ± 3 vs. WD 144 ± 2 mM; two‐way ANOVA age: *p* = .07, condition: *p* = .14, interaction: *p* = .48). Serum potassium and chloride concentrations were not different between age groups or conditions (*p* > .16 for all).

**TABLE 2:** Hydration measures

	Young		Older		*p*‐value (two‐way ANOVA)		
	Control	Water deprivation	Control	Water deprivation	Age	Condition	Interaction
Δ Body mass, %		−1 ± 2		−1 ± 1	.66	^a^	
Urine flow rate, L • 24 hr^−1^	1.0 ± 0.4	0.7 ± 0.1*	1.1 ± 0.3	0.7 ± 0.2*	.38	<0.01	0.62
24h urine osmolality, mOsm • kg H_2_O^−1^	437 ± 67	647 ± 86*	402 ± 62	599 ± 83*	.24	<0.01	0.74
24h urine specific gravity	1.012 ± 0.002	1.018 ± 0.002*	1.011 ± 0.002	1.016 ± 0.002*	.09	<0.01	0.31
Spot urine specific gravity	1.016 ± 0.007	1.023 ± 0.003*	1.011 ± 0.005	1.020 ± 0.004*	.16	<0.01	0.62
Plasma osmolality, mOsm • kg H_2_O^−1^	286 ± 2	288 ± 2	297 ± 5^#^	300 ± 4^#^	<.01	0.048	0.59
Thirst rating	3 ± 3	6 ± 2*	2 ± 2	6 ± 2*	.97	<0.01	0.43

Note

Data are presented as mean ± *SD*.

^a^Indicates unpaired, two‐tailed *t* test.

For post hoc analyses,^*^ indicates significant condition effect and ^#^ indicates significant age effect.

### 3.2 Cardiovascular measures during HG and PEI

**FIGURE 2:**
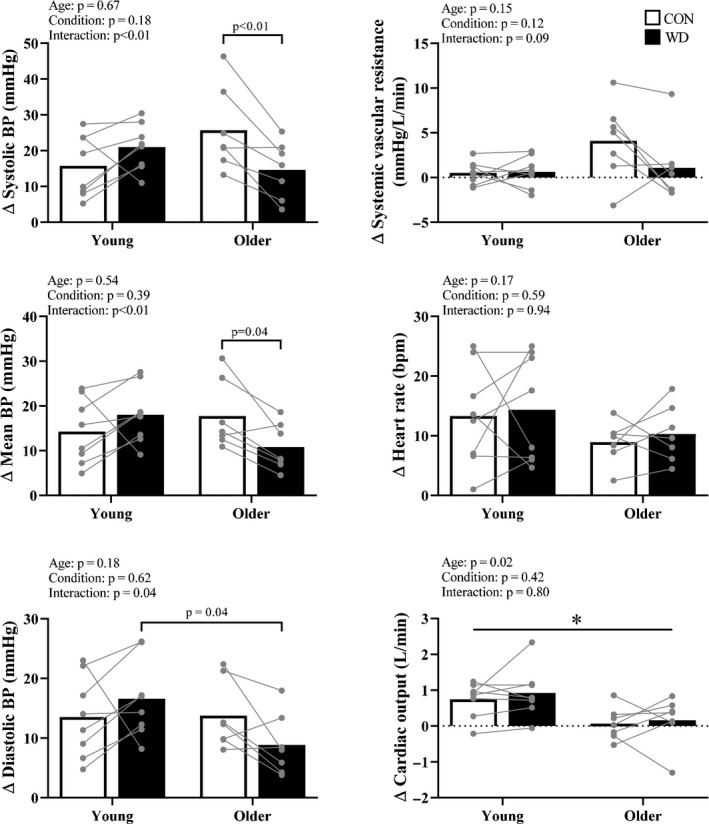
Handgrip exercise. Summary data with individual data points are presented for cardiovascular responses during the final minute of handgrip exercise (HG). Open bars indicate the euhydrated control condition (CON) and closed bars indicate the water deprived condition (WD).Systolic and mean blood pressure (BP) responses were lower during the WD in older adults and were not different between conditions in young adults. Diastolic BP responses were lower in old compared to young adults during WD. Systemic vascular resistance and heart rate responses during HG were not different between age groups or conditions. Young compared to older adults had augmented cardiac output responses. * indicates significant main effect of age

Systolic and mean BP responses during HG were lower during WD, compared to CON, in older adults and were not different between conditions in young adults (Figure 2). Diastolic BP responses during HG were lower in older compared to young adults during WD. SVR and heart rate responses during HG were not different between age groups or conditions. Young compared to older adults had augmented cardiac output responses during HG (Figure 2). Rating of perceived exertion (Borg 6–20) was not different between age groups or conditions (Young CON 15 ± 2 vs. WD 14 ± 3; Older CON 13 ± 4 vs. WD 13 ± 2; two‐way ANOVA age: *p* = .16, condition: *p* = .98, interaction: *p* = .76).

**FIGURE 3:**
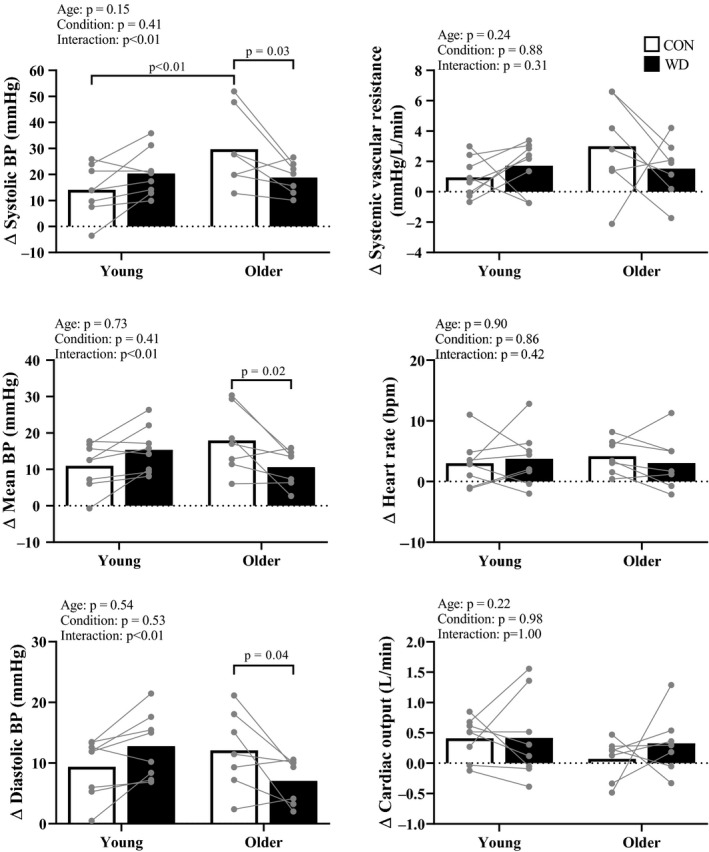
Post‐exercise ischemia. Summary data with individual data points are presented for cardiovascular responses during the final minute of post‐exercise ischemia (PEI). Open bars indicate the euhydrated control condition (CON) and closed bars indicate the water deprived condition (WD). Systolic blood pressure (BP) was greater in older compared to young adults during CON. Systolic, mean, and diastolic BP responses were lower in WD in older adults, and not different between conditions in young adults. Systemic vascular resistance, heart rate, and cardiac output responses were not different between age groups or conditions

Systolic BP responses during PEI were greater in older compared to young adults during CON. Systolic, mean, and diastolic BP responses during PEI were lower in WD in older adults, and not different between conditions in young adults. SVR, heart rate, and cardiac output responses during PEI were not different between age groups or conditions (Figure 3).

**FIGURE 4:**
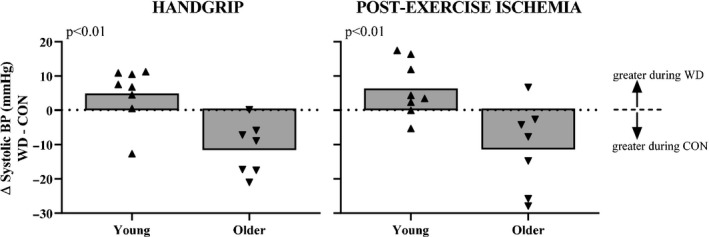
Age‐related differences in systolic blood pressure (BP) responses to handgrip exercise and post‐exercise ischemia after water deprivation (WD). Summary data (gray bars) with individual data points are presented for systolic BP responses (WD minus CON [euhydrated control condition]) during the final minute of handgrip exercise (left) and post‐exercise ischemia (right). These data indicate that the condition‐related differences in systolic BP responses were different between age groups. Specifically, the positive group mean for the young group suggests greater BP responses during WD whereas the negative group mean for the older group suggests greater BP responses during CON

There were divergent condition‐related differences (WD minus CON) in systolic BP responses during HG and PEI between age groups. All but one young participant demonstrated an increase in systolic BP responses during WD compared to CON. Conversely, all but one older participant demonstrated a decrease in systolic BP responses during WD compared to CON (Figure 4).

### 3.3 Cardiovascular measures during the CPT

**FIGURE 5:**
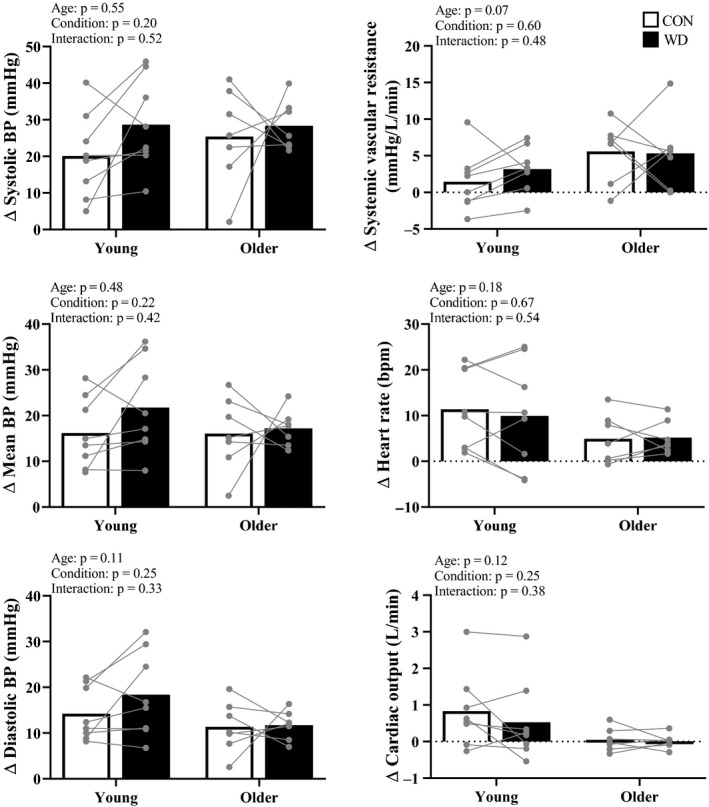
Cold pressor test. Summary data with individual data points are presented for cardiovascular responses during the final minute of the cold pressor test (CPT). Open bars indicate the euhydrated control condition (CON) and closed bars indicate the water deprived condition (WD). Systolic, mean, and diastolic blood pressure (BP) responses during the CPT were not different between age groups or conditions. Systemic vascular resistance, heart rate, and cardiac output responses were also not different between age groups or conditions

Systolic, mean, and diastolic BP responses during the CPT were not different between age groups or conditions. SVR, heart rate, and cardiac output responses during the CPT were also not different between age groups or conditions (Figure 5).

## 4 DISCUSSION

The primary novel findings of our study were that, (a) biochemical changes in urine and blood samples, as well as thirst ratings, were not different between healthy young and older female adults following short‐term WD, and (b) older female adults had attenuated pressor responses during isometric HG and PEI following short‐term WD. Additionally, as expected, older compared to young female adults had higher basal (i.e., CON) plasma osmolality values. Together, these data obtained from healthy young and older female adults contribute to our understanding of age‐related alterations in biochemical responses to acute reductions in water intake and the physiological factors that influence reflex BP regulation.

Our previous work (Watso et al., 2019) demonstrated that this model of reduced water intake effectively increases urine and plasma osmolality, urine specific gravity, and thirst, but does not augment exercise BP responses in healthy young adults. Together, these previous data suggested that this model of WD would be sufficient to examine our current hypothesis related to the influence of age on BP responses following reduced water intake. Consistent with our previous study, our present cohort completed the same protocol and demonstrated increased urine and plasma osmolality, urine specific gravity, and thirst following the WD condition, suggesting mild hypohydration.

We anticipated that the responses to WD would be exaggerated in older adults compared to young adults because of previous literature demonstrating lower basal total body water (Davy & Seals, 1985), altered extracellular fluid sensing (Phillips, Johnston, et al., 1993), blunted hormonal (e.g., antidiuretic hormone) release (Bevilacqua et al., 1987; Phillips, Bretherton, et al., 1993), and impaired kidney function (Crowe et al., 1987) in older adults. However, increases in plasma osmolality following WD were not different between age groups in our present study. One potential explanation is that much of this prior work was completed exclusively within male adults across the lifespan. Thus, those data may not necessarily extend to female adults during aging. More severe WD might be needed to elicit age‐related differences in the plasma osmolality response in female adults. However, the finding that older versus young female adults had higher basal plasma osmolality in the present study is consistent with published cross‐sectional data (Stookey, 2005). For the present study, it is unclear if this baseline difference between age groups could have influenced our findings. Interestingly, while inconsistent with previously published work in human participants, a recent study in rodents demonstrated attenuated increases in plasma osmolality in old (18 months) compared to young (three months) female rats following 48 hours of WD (Quiros Cognuck et al., 2020).

Prior work demonstrated that WD elevates sympathetic nerve activity to support BP (Brooks et al., 2004, 2005). However, we recently found that WD does not alter the reflex regulation of BP in rodents or healthy young adults. Specifically, we found that 48hours of WD in male rats did not exaggerate BP responses to the unilateral microinjection of glutamate (or GABA) in the rostral ventrolateral medulla (RVLM) or during the stimulation of sciatic afferents (Watso et al., 2019). Consistent with these findings in rodents, we demonstrated that short‐term WD does not increase exercise BP responses in healthy young male and female adults (Watso et al., 2019). Thus, the role of short‐term WD on exercise BP responses in older female adults was unclear. However, prior literature indicates that older compared to young male adults have greater increases in plasma osmolality/blood electrolyte concentrations following WD (Phillips et al., 1984), which could exaggerate BP responses during exercise. But, in contrast to this previous study that informed our hypothesis, the WD‐induced increase in plasma osmolality in our present study was not different between age groups. There are three major differences between that previous study and our current study. First, and most importantly, the participants in the previous study were all male whereas our research question was focused on female adults only. Second, it is unknown if the old adults in the previous study were sedentary or highly active whereas in our present study, habitual physical activity was similar between age groups. Third, while dietary status in the previous study was unclear, the participants in the present study maintained similar dietary sodium intake among both hydration conditions and age groups. Together, these reasons may explain why we did not find older adults to have larger increases in plasma osmolality following short‐term WD. Thus, the present investigation extends the findings of this previous work and provides new information for biochemical responses to short‐term WD in female aging.

Related to age‐related changes in reflex BP regulation, most (Boutcher & Stocker, 1999; Choi et al., 2012; Hartog et al., 2018; Lalande et al., 2014; Milia et al., 2015), but not all (Ng, Callister, Johnson, & Seals, 1994), previous work suggests greater exercise BP responses in older compared to younger adults. A more recent investigation matched young and older adults for physical activity status and reported that older female adults had greater systolic BP responses during static exercise than young female, young male, or older male adults (Trinity et al., 2018). Consistent with these previous reports, the mean values for increases in systolic BP were higher in older compared to young female adults for HG (post hoc *p* = .08) and PEI (post hoc *p* = .01) during the euhydrated control condition. Interestingly, we observed a significant interaction for systolic, mean, and diastolic BP responses during both HG and PEI. Specifically, systolic BP responses in older adults were significantly attenuated during WD compared to CON, whereas the mean values for systolic, mean, and diastolic BP responses in young adults were higher during WD compared to CON (seven out of eight participants had larger BP responses during WD compared to CON). Within our cohort, it is unclear why BP responses during exercise pressor reflex activation were attenuated in older female adults, while they were mildly hypohydrated. It is also unclear why BP values at rest were higher during experimentation compared to screening in older adults. For the latter, we would speculate that such observations in older adults could have resulted from postural differences (seated during screening versus supine during experimentation) and/or “white coat hypertension” as participants were instrumented with equipment to assess several other variables during experimentation (e.g., hydration markers in the blood via an intravenous catheter, beat‐to‐beat BP via photoplethysmography, etc.). While we do not think this resulted in a “ceiling” effect for BP, we cannot fully discount relatively higher resting systolic BP values during experimentation versus screening influencing exercise pressor responses (the primary variable of interest for this investigation) between experimental conditions.

Regarding BP responses during hypohydration, we previously found that short‐term WD does not augment BP or muscle sympathetic outflow responses during static exercise in healthy young male and female adults (Watso et al., 2019). Other work has reported moderate hypohydration (4 % body mass loss) to blunt mean BP responses during exercise with an ambient temperature of 40 °C, but not in ambient temperatures between 10 and 30 °C in young male adults (Kenefick, Sollanek, Charkoudian, & Sawka, 2014). Another study in young male adults reported similar BP responses during cycling exercise in mild heat (30 °C) following 24‐hr fluid restriction (Tankersley, Zappe, Meister, & Kenney, 1985). A more recent study in young male adults reported lower absolute mean BP during exercise following mild and moderate hypohydration (2 and 3.5 % body mass loss, respectively), but this study elicited hypohydration via exercise in the heat (37 °C) (Pearson et al., 2013). While heat stress was not used in the present study, we similarly found WD to attenuate BP responses during static exercise and PEI in older female adults. Related to the CPT, our finding that systolic BP responses were similar between age groups in female adults is consistent with two previous studies conducted in cohorts of male and female adults (Ng et al., 1994; Tonkin & Wing, 1994). The results from our study build on the published literature and provide evidence on the effect of reduced water intake on BP responses during exercise and the CPT in healthy female aging.

Finally, while the mechanisms mediating these age‐related changes are unclear, it is possible that the lack of estrogen after menopause reduces β‐adrenergic mediated vasodilation (which normally offsets α‐adrenergic mediated vasoconstriction) , subsequently enhancing SVR. In support of this, propranolol (β‐blocker) increased total peripheral resistance responses during ischemic HG in healthy young female adults, while attenuating cardiac output responses (Samora, Incognito, & Vianna, 1985). Therefore, it is possible that older adults, who have limited capabilities to increase cardiac output during exercise (Empel, Kaye, & Borlaug, 2014; Lalande et al., 2014; Ogawa et al., 1992; Trinity et al., 2018), must rely on SVR to raise BP and provide blood flow to active tissue. This is in agreement with previous studies reporting greater increases (or attenuated reductions) in SVR during exercise pressor reflex activation (Trinity et al., 2018) and metaboreflex isolation (Choi et al., 2012) in older compared to young female adults, potentially contributing to the augmented BP responses. Indeed, our present data demonstrate reduced cardiac output responses during HG in older compared to young female adults, regardless of condition. Thus, our data support the notion of age‐related reductions in cardiac output responses during exercise.

### 4.1 Limitations

While our study contributes new knowledge related to biochemical and exercise BP responses to short‐term WD in healthy female aging, there are several limitations to mention. First, the changes in hydration biomarkers between conditions were modest. However, we would posit that participants were euhydrated during CON and mildly hypohydrated during WD, as intended, for the following reasons: (a) reductions in body mass of 1 % fit within the criteria for mild hypohydration (McDermott et al., 2017), (b) the 24‐hour urine osmolality values > 500 mOsm • kg H2O^−1^ observed during WD indicate inadequate hydration status (Perrier et al., 2015), and (c) the absolute body mass values during screening were more similar to CON than WD. Further, despite the limited participants in this study, there were statistically significant differences between conditions for several urinary markers, plasma osmolality, and rating of thirst, indicating a changed hydration status. A second limitation would be related to our ability to detect significant effects of WD on BP responses during exercise. While our data were collected from a relatively small cohort (*n* = 15), the study was a randomized crossover design including two separate three‐day interventions and age groups were well matched for several physiological (e.g., non‐obese, etc.) and lifestyle (e.g., dietary sodium, habitual physical activity) factors. Further, significant interactions were observed for systolic, mean, and diastolic BP responses during HG exercise (our primary outcome variable) and for systolic, mean, and diastolic BP responses during PEI. Additionally, there was high consistency in statistical outcomes between groups for HG and PEI. Last, using the systolic BP responses data during HG, we calculated a post hoc effect size of 0.56, indicating that our achieved power for determining a significant within‐between interaction was 0.92, which surpasses the commonly used threshold of 0.80. A third limitation is that we do not know if there were alterations in muscle sympathetic nerve activity, plasma renin–angiotensin–aldosterone system hormones, or arginine vasopressin concentrations. However, the present results significantly contribute to our understanding of the physiological effects of reduced water intake and reflex BP regulation during female aging. Future studies are warranted to address the limitations discussed above.

### 4.2 Summary

The current data suggest that healthy female aging is associated with a maintained ability to preserve body water balance during short‐term reductions in water intake. These data also indicate that reduced water intake is not associated with augmented responses during exercise pressor reflex activation in older female adults.

## DISCLOSURES

The authors report no competing interests regarding the current article.

## AUTHORS' CONTRIBUTIONS

Experiments were performed in the Cardiovascular Physiology Research Laboratory at the University of Delaware. JCW, MCB, ATR, MAW, MMW, SDS, and WBF contributed to study design; all authors contribute to the acquisition, analysis, or interpretation of data; JCW drafted the manuscript and all authors revised it critically for important intellectual content. All authors approved the final version of the manuscript. All authors agree to be accountable for all aspects of the work in ensuring that questions related to the accuracy or integrity of any part of the work are appropriately investigated and resolved. All persons designated as authors qualify for authorship, and all those who qualify for authorship are listed.

5

**TABLE 3:** Resting cardiovascular measures

	Young		Older		*p*‐value (Two‐way ANOVA)		
	Control	Water deprivation	Control	Water deprivation	Age	Condition	Interaction
*Handgrip exercise trial baseline*							
Systolic BP, mmHg	108 ± 6	109 ± 8	150 ± 21^#^	140 ± 19^#^	<.01	0.20	0.12
Mean BP, mmHg	79 ± 4	80 ± 5	102 ± 11^#^	97 ± 11^#^	<.01	0.32	0.12
Diastolic BP, mmHg	64 ± 4	65 ± 5	78 ± 7^#^	76 ± 9^#^	<.01	0.67	0.19
Systemic vascular resistance, mmHg • cardiac output ^−1^	17 ± 4	17 ± 4	25 ± 5^#^	24 ± 6^#^	<.01	0.76	0.56
Cardiac output, L • min ^−1^	5 ± 1	5 ± 1	4 ± 1	4 ± 1	.12	0.89	0.89
Stroke volume, mL	80 ± 15	82 ± 16	68 ± 16	70 ± 9	.09	0.59	0.84
Heart rate, beats • min ^−1^	62 ± 4	60 ± 5	61 ± 10	61 ± 12	.87	0.42	0.53
*Cold pressor test baseline*							
Systolic BP, mmHg	109 ± 6	110 ± 9	151 ± 20^#^	141 ± 21^#^	<.01	0.18	0.06
Mean BP, mmHg	79 ± 5	80 ± 6	103 ± 12^#^	98 ± 13^#^	<.01	0.21	0.08
Diastolic BP, mmHg	64 ± 5	65 ± 6	79 ± 9^#^	76 ± 10^#^	<.01	0.31	0.20
Systemic vascular resistance, mmHg • cardiac output ^−1^	17 ± 4	17 ± 3	26 ± 3^#^	25 ± 8^#^	<.01	0.47	0.80
Cardiac output, L • min ^−1^	5 ± 1	5 ± 1	4 ± 1	4 ± 1	.04	0.46	0.87
Stroke volume, mL	79 ± 19	81 ± 15	66 ± 12	68 ± 8	.09	0.43	0.85
Heart rate, beats • min ^−1^	62 ± 8	62 ± 6	61 ± 9	61 ± 12	.79	0.90	0.95

Note

Data are presented as mean ± *SD*. For post hoc analyses, ^#^ indicates a significant age effect. BP, brachial artery blood pressure.
